# Venous Ischemic Pancreatitis With Reduced Pancreatic Enzymes Secondary to Extensive Splanchnic Venous Thrombosis: An Unrecognized Vascular Phenotype

**DOI:** 10.7759/cureus.106237

**Published:** 2026-03-31

**Authors:** Niyas Khalid Ottu Para, Khaled Ghorab, Ahmed Ghorab

**Affiliations:** 1 Internal Medicine, Burjeel Hospital, Abu Dhabi, ARE; 2 Adult Intensive Care, Burjeel Hospital, Abu Dhabi, ARE; 3 Medicine, East Lancashire NHS Hospitals Trust, Blackburn, GBR

**Keywords:** bariatric surgery-associated thrombosis, ischemic pancreatic injury, portal vein thrombosis, splanchnic venous thrombosis, splenic vein thrombosis, vascular pancreatitis, venous ischemic pancreatitis

## Abstract

Acute pancreatitis is commonly diagnosed on the basis of characteristic abdominal pain, elevation of pancreatic enzymes to greater than three times the upper limit of normal, and supportive imaging findings. However, pancreatic injury may occasionally arise from vascular mechanisms rather than the classical enzyme-mediated inflammatory process. Venous ischemic pancreatitis, secondary to splanchnic venous thrombosis, is an under-recognized clinical entity that may present with normal or even reduced pancreatic enzyme levels, creating a potential diagnostic challenge.

We report the case of a 49-year-old woman who presented with severe abdominal pain and radiologic features consistent with pancreatic injury despite both amylase and lipase levels being below the normal reference range. Contrast-enhanced computed tomography (CT) demonstrated a bulky pancreas with areas of hypoenhancement along with extensive thrombosis of the splenic vein, portal vein, and superior mesenteric vein. CT angiography confirmed preserved arterial inflow, supporting venous outflow obstruction as the dominant mechanism of pancreatic ischemia. Comprehensive evaluation excluded common causes of pancreatitis, including gallstone disease, alcohol-related pancreatitis, malignancy, myeloproliferative neoplasms, antiphospholipid syndrome, and major inherited thrombophilias. Therapeutic anticoagulation was initiated promptly, resulting in rapid clinical improvement and preventing progression to bowel ischemia or pancreatic necrosis.

This case highlights an under-recognized vascular phenotype of pancreatic injury consistent with venous ischemic pancreatitis and illustrates how careful integration of biochemical findings, vascular imaging, and clinical reasoning can guide timely anticoagulation and dramatically alter clinical outcomes.

## Introduction

Acute pancreatitis is a common cause of acute abdominal pain requiring hospitalization. It is classically driven by acinar cell injury with premature intrapancreatic activation of digestive enzymes, leading to local pancreatic inflammation and systemic inflammatory responses [1]. In routine clinical practice, diagnosis relies on a combination of characteristic abdominal pain, elevation of serum pancreatic enzymes, and supportive imaging findings, as reflected in the revised Atlanta classification and current international consensus guidelines [1]. Consequently, measurement of serum amylase and lipase has become a central component of the early diagnostic evaluation of patients presenting with suspected pancreatitis, though up to 20% of cases may present with normal enzyme levels, particularly in alcoholic or late-presenting pancreatitis.

Despite this well-established paradigm, pancreatic injury may also arise through mechanisms that are not primarily enzyme-mediated. One such mechanism is ischemic pancreatic injury, which occurs when pancreatic perfusion is compromised by vascular disturbances such as systemic hypotension, arterial occlusion, or microcirculatory dysfunction [2]. In these situations, pancreatic damage results from cellular hypoxia and impaired microvascular perfusion rather than classical enzyme-driven inflammation. Because ischemia suppresses acinar metabolic activity and enzyme synthesis, serum pancreatic enzyme levels may remain normal or even reduced despite significant pancreatic injury, a phenomenon documented in experimental models of ischemic pancreatitis [2]. While normoamylasemic pancreatitis is a recognized entity, the presence of reduced enzyme levels in this case suggests a distinct mechanism related to ischemia-driven pathophysiological mechanism characterized by suppression of pancreatic exocrine function. This biochemical-radiologic dissociation represents an important but under-recognized diagnostic challenge and may lead clinicians to prematurely exclude pancreatic pathology.

An even less commonly recognized mechanism of pancreatic injury is venous ischemia secondary to splanchnic venous outflow obstruction. While acute pancreatitis is well known to predispose to splenic and portal vein thrombosis as a secondary complication, the reverse relationship--extensive splanchnic venous thrombosis resulting in venous congestion and pancreatic ischemia--has been described only sporadically in the literature [3]. In such cases, impaired venous drainage leads to increased intrapancreatic venous pressure, reduced capillary perfusion, and subsequent ischemic injury to pancreatic tissue despite preserved arterial inflow.

Failure to recognize this vascular phenotype has important clinical implications. When pancreatic enzyme levels are normal or reduced, clinicians may prematurely exclude pancreatitis and pursue alternative diagnoses, potentially delaying anticoagulation or exposing patients to unnecessary invasive interventions. Awareness of venous ischemic pancreatitis, therefore, requires careful integration of clinical presentation, biochemical findings, and vascular imaging.

In this report, we describe a patient who presented with severe abdominal pain and radiologic features of pancreatitis despite reduced pancreatic enzyme levels and was ultimately found to have extensive splenic, portal, and superior mesenteric venous thrombosis with preserved arterial inflow. The case highlights an under-recognized vascular phenotype of pancreatic injury consistent with venous ischemic pancreatitis and illustrates how early recognition and prompt anticoagulation can lead to rapid clinical recovery and prevention of serious complications.

## Case presentation

A 49-year-old Emirati woman presented with the acute onset of severe abdominal pain. She described severe, persistent abdominal pain predominantly in the epigastric region, with radiation to the back. The pain was not clearly related to food intake and was associated with progressive discomfort and functional limitation. She denied alcohol consumption, recent trauma, gallstone disease, or prior episodes of pancreatitis. Her past medical history was notable for prior bariatric surgery. On examination, she was hemodynamically stable but appeared in significant distress due to abdominal pain.

Initial laboratory evaluation revealed a normal white blood cell count with elevated inflammatory markers. Total bilirubin was mildly elevated, while coagulation parameters, including prothrombin time and international normalized ratio, were within normal limits. Notably, serum pancreatic enzymes were not elevated; both amylase (22 U/L) and lipase (18 U/L), measured within 24 hours of presentation, were below the normal reference range (see Table 1). Serum calcium, triglycerides, and CA 19-9 levels were within normal limits. Serial laboratory evaluation during hospitalization demonstrated persistently low pancreatic enzyme levels despite radiologic evidence of pancreatic injury. The temporal evolution of biochemical parameters during and after the episode is summarized in Table 1.

**Table 1:** Key laboratory investigations during hospitalization and follow-up. Selected laboratory parameters during the acute presentation and follow-up.

Parameter	Admission	Peak/Early hospitalization	Discharge	Follow-up	Reference range
C-reactive protein (mg/L)	59.9	57.8	23.1	4.1	<5
Total bilirubin (µmol/L)	24.8	20.3	17.5	Normal	<20
Direct bilirubin (µmol/L)	12.2	9.7	7.6	Normal	<5
Lipase (U/L)	18	20	24	29	0-60
Amylase (U/L)	22	26	32	37	28-100
Fecal elastase (µg/g stool)	276	-	-	267	>200
Creatinine (µmol/L)	48	46	53	55	45-90
Sodium (mmol/L)	139	138	140	142	135-145
Potassium (mmol/L)	3.9	3.7	4.2	4.3	3.5-5
D-dimer (mg/L)	3.0	3.4	0.7	0.2	<0.5

**Figure 1:**
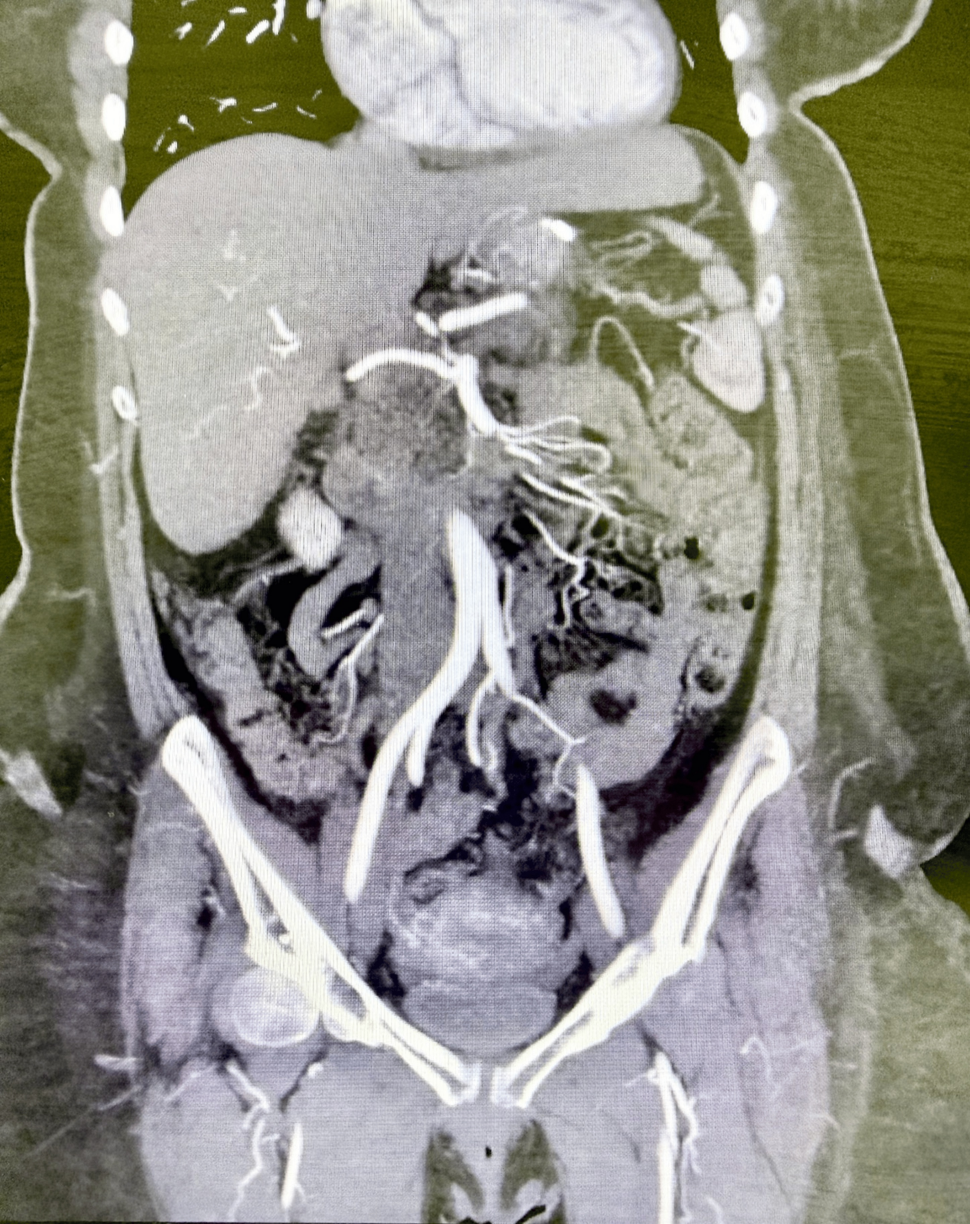
Contrast-enhanced CT abdomen demonstrating venous thrombosis with pancreatic involvement. Coronal contrast-enhanced CT image demonstrating thrombosis of the mesenteric venous system with surrounding mesenteric congestion and inflammatory changes involving the pancreas. CT: computed tomography.

**Figure 2:**
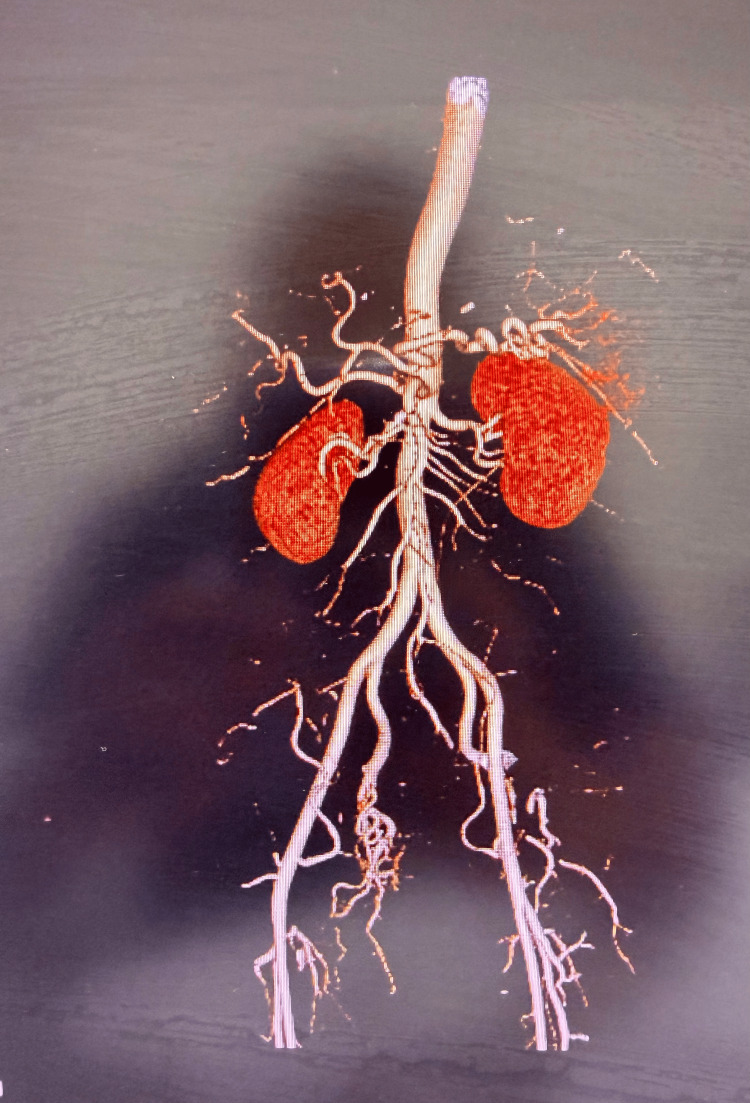
Three-dimensional CT angiographic reconstruction of the abdominal vasculature. Three-dimensional CT angiographic reconstruction demonstrating the abdominal aorta and major visceral arterial branches, including the celiac axis, superior mesenteric artery, and renal arteries with preserved arterial patency. CT: computed tomography.

Contrast-enhanced computed tomography (CT) performed 24 hours after symptom onset demonstrated a bulky pancreas with indistinct margins and surrounding fat stranding. Focal hypoenhancement was observed involving the pancreatic head and body, without evidence of pancreatic duct dilatation or peripancreatic fluid collections. In addition, extensive thrombosis of the splenic vein, portal vein, and superior mesenteric vein was identified (Figure 1). Mild splenomegaly and minimal ascites were also present. To further characterize the vascular findings, CT angiography was performed. This demonstrated preserved arterial inflow through the celiac axis and superior mesenteric artery, with normal enhancement of the arterial vasculature, confirming that arterial perfusion was intact (Figure 2). In contrast, the splenic vein, portal vein, and superior mesenteric vein were distended and filled with thrombus, consistent with extensive acute splanchnic venous thrombosis and venous outflow obstruction, without evidence of cavernous transformation to suggest chronicity. Bowel perfusion was preserved, and no evidence of bowel wall thickening or intestinal ischemia was observed.

A comprehensive etiological evaluation was undertaken to identify potential causes of pancreatitis and thrombosis. Gallstone disease, pancreatic malignancy, autoimmune pancreatitis, and other structural pancreatic abnormalities were excluded. Evaluation for thrombophilia revealed normal protein C, protein S, antithrombin activity, and homocysteine levels. Antiphospholipid antibody testing, including anticardiolipin IgG and IgM antibodies, was negative. Screening for myeloproliferative neoplasms was also negative, with absence of both JAK2 V617F and MPL mutations. The coagulation profile remained normal. Tumor marker evaluation showed normal CA 19-9 levels (9 U/mL). Additional autoimmune screening, including rheumatoid factor and anticyclic citrullinated peptide antibodies, was negative. Genetic testing demonstrated homozygosity for the MTHFR A1298C variant; however, homocysteine levels were within normal limits, and this finding is not considered to have a significant independent thrombogenic role. The results of the etiological evaluation are summarized in Table 2.

**Table 2:** Etiologic evaluation for pancreatitis and thrombophilia. Investigations performed to exclude common causes of pancreatitis and underlying prothrombotic conditions.

Investigation	Result	Interpretation		
Triglycerides	Normal	Hypertriglyceridemia excluded		
Serum calcium	Normal	Hypercalcemia excluded		
CA 19-9	9 U/mL	Pancreatic malignancy unlikely		
Anticardiolipin IgG	Negative	Antiphospholipid syndrome excluded		
Anticardiolipin IgM	Negative	Antiphospholipid syndrome excluded		
Protein C activity	Normal	Inherited thrombophilia unlikely		
Protein S activity	Normal	Inherited thrombophilia unlikely		
Antithrombin III activity	Normal	Inherited thrombophilia unlikely		
JAK2 mutation	Negative	Myeloproliferative neoplasm excluded		
MPL mutation	Negative	Myeloproliferative neoplasm excluded		
MTHFR mutation	Homozygous A1298C	Possible thrombotic risk modifier		

Given the presence of extensive splanchnic venous thrombosis, with imaging findings consistent with pancreatic injury and absence of typical causes of inflammatory pancreatitis, venous ischemic pancreatic injury was suspected. Therapeutic anticoagulation with low-molecular-weight heparin was initiated promptly, and a thrombophilia workup was performed subsequently. Following initiation of anticoagulation, the patient demonstrated rapid clinical improvement. Abdominal pain progressively resolved over the subsequent days, and inflammatory markers improved. She was transitioned to oral anticoagulation prior to discharge and advised to have close follow-up.

Subsequent outpatient follow-up demonstrated continued clinical stability, with complete resolution of abdominal pain and no recurrence of thrombotic or pancreatic events while continuing anticoagulant therapy. Repeat laboratory evaluation showed normalization of serum amylase levels following recovery. Importantly, fecal elastase levels remained within the normal range both before and after the episode, indicating preserved exocrine pancreatic function and suggesting that early restoration of venous outflow prevented irreversible pancreatic injury.

## Discussion

This case underscores several important diagnostic and therapeutic considerations in patients presenting with abdominal pain and imaging findings suggestive of pancreatic injury. A major diagnostic pitfall in this scenario is reliance on pancreatic enzyme levels to exclude pancreatitis. In classical inflammatory pancreatitis, acinar cell injury results in leakage of digestive enzymes into the circulation, leading to elevation of serum amylase and lipase levels. Lipase, owing to its longer half-life and greater pancreatic specificity, is considered the more reliable diagnostic marker, whereas amylase demonstrates more rapid clearance and lower specificity. In contrast, ischemic pancreatic injury is characterized by cellular hypoxia, mitochondrial dysfunction, and early suppression of enzyme synthesis and secretion, which may result in normal or even reduced pancreatic enzyme levels despite significant parenchymal injury, as observed in the present case [2,4]. This biochemical-radiologic dissociation has been described in ischemic pancreatic injury and should prompt clinicians to reconsider enzyme-centric diagnostic paradigms when clinical presentation and imaging findings are discordant. This case highlights venous outflow obstruction as an under-recognized vascular mechanism of pancreatic injury that may mimic inflammatory pancreatitis while demonstrating an atypical biochemical profile characterized by reduced pancreatic enzyme levels.

Although ischemic pancreatic injury has been described in the setting of systemic hypotension, arterial occlusion, or postoperative states, venous mechanisms of pancreatic ischemia remain less frequently recognized. Importantly, formal diagnostic criteria for ischemic pancreatitis have not been established. Most reports, therefore, rely on a clinicoradiologic framework integrating imaging evidence of pancreatic injury, identification of a vascular event capable of compromising pancreatic perfusion, exclusion of common etiologies of pancreatitis, and demonstration of preserved arterial inflow when venous mechanisms are suspected. In the present case, the combination of imaging findings consistent with pancreatic injury, extensive splanchnic venous thrombosis with preserved arterial perfusion, absence of traditional pancreatitis risk factors, reduced pancreatic enzyme levels, and rapid clinical improvement following anticoagulation collectively supported the diagnosis of venous ischemic pancreatic injury.

A second important diagnostic consideration involves the directionality of the relationship between pancreatitis and splanchnic venous thrombosis. Acute pancreatitis is a well-recognized cause of secondary portal and splenic vein thrombosis due to local inflammatory changes, endothelial injury, and vascular compression. However, extensive multisegment splanchnic venous thrombosis in the absence of established pancreatitis risk factors should raise suspicion for a primary thrombotic event rather than a secondary complication [5]. In the present case, the extent of venous involvement, the presence of preserved arterial inflow, the early imaging finding of pancreatic hypoenhancement, and the absence of significant peripancreatic fluid collections or necrosis strongly supported venous congestion and ischemia as the initiating pathophysiologic mechanism rather than a downstream consequence of inflammatory pancreatitis.

CT angiography played a pivotal role in refining the diagnosis. Demonstration of intact arterial inflow effectively excluded arterial ischemia and mesenteric occlusive disease, thereby narrowing the underlying mechanism to venous outflow obstruction [3]. This distinction is clinically important because management strategies differ significantly. Arterial mesenteric ischemia may require urgent revascularization or surgical intervention, whereas venous ischemia resulting from splanchnic venous thrombosis is primarily managed with anticoagulation and supportive care [6]. Anticoagulation in splanchnic venous thrombosis is not universally standardized; however, current evidence and expert consensus generally support treatment for at least three to six months, with consideration of extended therapy in cases with extensive thrombosis or persistent risk factors. Follow-up in such cases typically includes clinical assessment and interval imaging to evaluate thrombus resolution and vascular patency, although standardized monitoring protocols remain variable. Long-term recurrence risk remains incompletely defined in cases of splanchnic venous thrombosis, highlighting the need for individualized duration of anticoagulation and follow-up.

Another notable feature of this case is the patient’s history of prior bariatric surgery. Increasing evidence suggests that post-bariatric physiology may predispose patients to alterations in splanchnic hemodynamics, episodic dehydration, and metabolic fluctuations that contribute to a prothrombotic environment [7]. Porto-mesenteric venous thrombosis has been reported as an uncommon but recognized complication following bariatric procedures. Proposed mechanisms include postoperative hypercoagulability, relative dehydration, rapid weight loss, and changes in abdominal pressure and venous flow dynamics [8,9]. Although such complications are most frequently reported in the early postoperative period, physiological changes associated with bariatric surgery may persist and potentially increase thrombotic susceptibility in predisposed individuals. In the present patient, the history of two prior bariatric procedures may therefore have contributed to an underlying prothrombotic milieu.

Genetic testing in this patient demonstrated homozygosity for the MTHFR A1298C variant. While MTHFR polymorphisms alone are not generally considered major inherited thrombophilic disorders, they may contribute to thrombotic susceptibility in the presence of additional genetic, metabolic, or environmental risk factors [10]. In this context, the MTHFR variant may have acted as a permissive risk modifier rather than a primary cause of thrombosis, particularly when combined with physiological changes associated with prior bariatric surgery.

The patient’s rapid and sustained clinical improvement following initiation of anticoagulation further supports venous ischemia as the dominant pathophysiologic mechanism. Reduction of venous congestion likely restored pancreatic microcirculatory perfusion, limited progression of ischemic injury, and prevented complications such as bowel ischemia, portal cavernoma formation, or pancreatic necrosis [5,6]. This clinical response highlights anticoagulation as both a therapeutic and potentially diagnostic intervention in selected patients with suspected venous ischemic pancreatitis.

An additional observation supporting the ischemic mechanism in this case was the preservation of exocrine pancreatic function. Fecal elastase levels remained within the normal range both before and after the episode, suggesting that early restoration of venous outflow prevented irreversible acinar injury. However, fecal elastase may remain normal even with moderate parenchymal loss due to functional reserve, so normal levels do not completely exclude subclinical injury. Furthermore, the subsequent normalization of serum amylase levels following clinical recovery is consistent with transient suppression of acinar enzyme production during the ischemic phase rather than destructive inflammatory pancreatitis.

Taken together, this case illustrates how integration of biochemical findings, vascular imaging, and clinical context can reveal an uncommon vascular mechanism of pancreatic injury. Recognition of this entity is particularly important because the therapeutic approach differs fundamentally from that of classical inflammatory pancreatitis. Early identification of venous ischemic pancreatic injury and prompt initiation of anticoagulation may lead to rapid clinical recovery and prevent potentially catastrophic complications associated with untreated splanchnic venous thrombosis. Similar diagnostic challenges have been described in other vascular abdominal pathologies, where nonspecific clinical presentation and initially inconclusive biochemical findings may delay diagnosis and appropriate management. This underscores the importance of maintaining a high index of suspicion for vascular etiologies in patients with discordant clinical, biochemical, and imaging findings. Clinicians should therefore consider venous ischemic pancreatic injury in patients presenting with abdominal pain and imaging findings suggestive of pancreatitis despite normal or reduced pancreatic enzyme levels, particularly when extensive splanchnic venous thrombosis is identified on vascular imaging.

## Conclusions

Venous ischemic pancreatic injury represents an uncommon but clinically important vascular mechanism of pancreatic pathology that may present with imaging findings suggestive of pancreatitis despite normal or reduced pancreatic enzyme levels. Extensive splanchnic venous thrombosis should prompt consideration of venous congestion and pancreatic ischemia, particularly when conventional etiologies of inflammatory pancreatitis are absent. In such cases, preserved arterial inflow on vascular imaging may provide an important clue to the underlying mechanism of pancreatic injury.

This case highlights the importance of integrating biochemical findings, vascular imaging, and clinical context when evaluating atypical presentations of pancreatic injury. Recognition of this vascular phenotype is particularly important because the therapeutic approach differs from that of classical inflammatory pancreatitis. Early recognition of this vascular phenotype and prompt initiation of anticoagulation may lead to rapid clinical recovery, preservation of pancreatic function, and prevention of potentially serious complications associated with untreated splanchnic venous thrombosis. This case also illustrates a distinct clinicoradiologic pattern in which pancreatic injury may occur secondary to venous outflow obstruction despite minimal biochemical evidence of pancreatitis. This case supports the expansion of current diagnostic frameworks beyond enzyme-based criteria in suspected pancreatic pathology.
